# Cultural events – does attendance improve health? Evidence from a Polish longitudinal study

**DOI:** 10.1186/s12889-016-3433-y

**Published:** 2016-08-05

**Authors:** Dorota Węziak-Białowolska, Piotr Białowolski

**Affiliations:** 1 European Commission, Joint Research Centre (JRC), Directorate I. Competences, Modelling, Indicators and Impact Evaluation Unit, Via E. Fermi 2749, 21027 Ispra (VA), Italy; 2 Department of Economics, Social Studies, Applied Mathematics and Statistics, University of Turin, corso Unione Sovietica 218bis, 10134 Torino, Italy

**Keywords:** Cultural attendance, Self-reported health, Patient health questionnaire, Longitudinal data, Causal analysis

## Abstract

**Background:**

Although there is strong advocacy for uptake of both the arts and creative activities as determinants of individual health conditions, studies evaluating causal influence of attendance at cultural events on population health using individual population data on health are scarce. If available, results are often only of an associative nature. In this light, this study investigated causative impact of attendance at cultural events on self-reported and physical health in the Polish population.

**Methods:**

Four recent waves (2009, 2011, 2013 and 2015) of the biennial longitudinal Polish household panel study, Social Diagnosis, were analysed. The data, representative for the Polish population aged over 16, with respect to age, gender, classes of place of residence and NUTS 2 regions, were collected from self-report questionnaires. Causative influence of cultural attendance on population health was established using longitudinal population representative data. To account for unobserved heterogeneity of individuals and to mitigate issues caused by omitted variables, a panel data model with a fixed effects estimator was applied. The endogeneity problem (those who enjoy good health are more likely to participate in cultural activities more frequently) was circumvented by application of instrumental variables.

**Results:**

Results confirmed positive association between cultural attendance and self-reported health. However, in contrast to the often suggested positive causative relationship, such a link was not confirmed by the study. Additionally, no evidence was found to corroborate a positive impact from cultural attendance on physical health. Both findings were substantiated by augmentation in the longitudinal perspective and causal link.

**Conclusions:**

We showed the relation between attendance at cultural events and self-reported health could only be confirmed as associational. Therefore, this study provided little justification to encourage use of passive cultural participation as a measure of health promotion (improvement). Our study did not confirm any identifiable benefit to physical health from passive participation in culture. Future research should investigate the causative influence of active participation in creative activities on health outcomes as, in contrast to passive attendance, it may be influential.

## Background

There is a common belief that engagement with the arts and cultural activities is associated with improvement of well-being and contributes to better quality of life. Support for this can be found from research using arts and creative activity therapies in clinical settings for a range of physical and mental health conditions. Although these therapies improved outcomes and prognosis for survival [1–3], association between positive behavioural and social changes and cultural participation, to maintain good or prevent ill health, should be interpreted with caution. Even though research has demonstrated such a relationship [3–5], the findings are mostly based on cross-sectional data or associational analysis. This implies that any relationship they report may be spurious, which casts doubt on the existence of a causal relationship.

Despite strong advocacy for [6], and extensive uptake of, the arts and creative activities as determinants for mental [1], physical [7] and self-reported health [5], as well as for conveying health promotion messages [3, 8], to the best of our knowledge there has been no evaluation of any causative influence of engagement with the arts or similar creative activities on population health with individual population data. Scarcity of longitudinal panel data simultaneously investigating health and cultural engagement and the challenges to establishing causality may present a bar to a more profound understanding of the nature of the relationship. The commonly reported, positive relationship between the two (as in the recently published in the BMC Public Health journal paper of Hansen et al. [3]) may result from the omission of unobserved individual level factors from cross-sectional (often linear or logit/probit regression) analysis or – more importantly – the phenomenon of reverse causality. Both these issues introduce endogeneity, rendering results implausible, since they do not accommodate the problem and, therefore, disallow valid conclusion. In the presence of endogeneity, any positive influence from cultural attendance on individual health status may appear causal but ensue from the simple fact that healthier people are more likely to attend cultural events.

Therefore, the aim of this study was to assess the causative relationship of cultural attendance on self-reported and physical health in the Polish population aged over 16. It offers additional insight into the issue of the relationship between cultural attendance and health, which, to the best of our knowledge, had only been proved, so far, to be associative. Two hypotheses were tested. The first, that more frequent cultural event participation had a positive causal influence on self-reported health (H1) and the second, that more frequent cultural event participation decreased prevalence of ill-health (H2). To the best of our knowledge, this is the first study to exploit longitudinal panel data to examine causality at individual level. Further, benefiting from the application of instrumental variables, the study circumvented the problems of endogeneity and potential reverse causality.

In the following sections, we first present the data and methods applied to examine the causality between cultural participation and health. Then, the selection of instruments is described followed by results obtained. The final section provides a concluding summary of findings with explanation of limitations of the study.

## Methods

### Data source

Analysis builds on four recent waves of the biennial Polish household panel study (2009, 2011, 2013 and 2015) – Social Diagnosis [9, 10]. Social Diagnosis is an observational longitudinal study aimed at investigating living conditions and the well-being of Poles. The data are representative for the Polish population aged over 16, with respect to age, gender, classes of place of residence and NUTS 2^1^ regions. Data on cultural attendance, self-reported health and ill-health, as well as socio-economic characteristics were collected by self-report questionnaires. The individual-level data and survey documentation are freely available in the public domain (http://www.diagnoza.com).

As the goal was to investigate a causal link between the cultural attendance and health outcomes, availability of the longitudinal dataset was of critical importance. Application of the statistical longitudinal modelling techniques, contrary to analyses conducted on cross-sectional data, offered more reliable causal findings by virtue of depiction of the logical temporal sequence of cause and effect, clear in the data.

### Measures

#### Cultural participation

Attendance at cultural events was assessed by the single question: How often have you been to the movies, play/theater or/and have you gone to a concert in the last month? Respondents reported the total number of cultural events attended. From these figures, culture attendance was measured as a continuous variable in the analysis (Mean_2009_ = 0.33, SD_2009_ = 0.84, Min_2009_ = 0, Max_2009_ = 20; Mean_2011_ = 0.34, SD_2011_ = 1.07, Min_2011_ = 0, Max_2011_ = 30; Mean_2013_ = 0.34, SD_2013_ = 0.96, Min_2013_ = 0, Max_2013_ = 30; Mean_2015_ = 0.35, SD_2015_ = 0.94, Min_2015_ = 0, Max_2015_ = 25).

#### Outcome variables: health

Two complementary outcomes were considered: self-reported health (SRH) and medical problems, which were reflected by a count of somatic symptoms in the month preceding the survey. SRH is measured on a 6-point Likert scale (6-extremely satisfied, 5-very satisfied, 4-somewhat satisfied, 3-somewhat dissatisfied, 2-very dissatisfied, 1-extremely dissatisfied). Somatic symptoms were measured using the Patient Health Questionnaire (PHQ-15, see: Appendix) [11, 12]. The scale ranged from 0 to 30 and was represented in the analysis as a continuous variable (Mean_2009_ = 5.03, SD_2009_ = 4.78; Mean_2011_ = 4.83, SD_2011_ = 4.67; Mean_2013_ = 4.68, SD_2013_ = 4.62; Mean_2015_ = 4.47, SD_2015_ = 4.53). The PHQ-15 scale, used to assess occurrence and severity of common somatic symptoms, is one of the most widely applied and best-validated self-report measures for somatic symptom burden [13].

#### Control variables

Health depends on many factors including gender, age, educational level, employment status and income [14, 15]. All these factors were reflected in the study. However, gender was regarded as time-invariant and not analysed in the longitudinal setting, in the fixed effect panel regression. Age and education level reflected the number of exact years lived and spent in education and were applied to the analysis as continuous variables, together with income after log transformation. Labour market status was measured as a dichotomous variable with unemployment was coded as a distinct status, justified by previous studies which identified it as a significant stressor to health [16, 17].

### Panel attrition

In 2009, the study covered 9241 individuals aged 16 and over. Of these participants, 8420 individuals were then surveyed in 2011 along with 4930 new respondents who entered the panel. In 2013, 11,650 individuals returned from the 2011 study and 4136 new respondents joined. And finally, of the 2013 participants, in 2015 12,555 respondents returned and a further 1030 new respondents joined. Therefore, 5443 individuals participated in all four waves, 3486 participated in three waves and 1707 were participants in only two waves. For this analysis, individuals who participated in only one wave were excluded as longitudinal analysis requires multiple participation.

To deal with selective panel attrition, inverse probability-of-attrition weight was applied to each observation, as proposed by [18]. The property of these weights is to up-weight respondents with characteristics similar to those of respondents missing due to attrition. To compute weights, we used pooled logistic regression to compute probability of attrition due to respondent dropout between waves. As the main concern was to examine selectivity of panel attrition with respect to the relationship examined (i.e., influence of cultural attendance on health), both cultural attendance and health outcome were introduced to the logistic model along with all control variables (described in following subsection). As two different health outcomes were investigated, two logistic regression models – differing with respect to health variable – were run providing two sets of attrition weights for further analysis (detailed results available upon request).

Additionally, all individuals with missing values on health, control or instrumental variables were excluded from analysis, i.e., within-wave missing values. However, to check robustness of our results, the multiply imputed data set (10 imputations) was reanalysed. Data were arranged in a wide structure as suggested by [19], but this approach was not computationally feasible, problem reported as common with longitudinal data [20]. Therefore, multiple imputations were performed on a long formatted data set (shown by [20] to be only slightly inferior). However, at this time, it was not computationally possible to accommodate multiple imputations in an advanced IV setting, as Rubin’s formula [21] does not permit identification tests for IV, therefore only results for OLS and fixed effects could be obtained. These estimates (see: Appendix: Tables 4 and 5, column ‘*No weighs, multiple imputations (10)*’) did not vary, either with respect to sign or significance from those presented in the following section, thus proving their robustness.

### Statistical methods

The relation between cultural attendance and health was modelled as follows:

$$ healt{h}_{it}={\beta}_0+{\beta}_1{X}_{it}+{\gamma}_1C{A}_{it}+{\eta}_{it},i=1,\dots, N;t=1,\dots, T $$

where subscript *i* is for the individual, *t* is for time, *X*_*it*_ is a vector of control variables, *CA*_*it*_ is a cultural attendance indicator, *η*_*it*_ is a disturbance term; *health*_*it*_ is either SRH or PHQ-15 as two independent models are estimated; *β*_*0*_ represents a constant, *β*_*1*_ shows the individual level characteristics (control variables) on health outcome and *γ*_*1*_ shows the effect of cultural attendance on health outcome. The relationship was modelled using the inverse probability-of-attrition weights – as described in the previous subsection.

In the first step, to check association between active cultural activity and health outcomes the simple ordinary-least-squares (OLS) regression was run. It should be noted, however, that standard OLS estimates could yield unbiased results, only when the hypothesis of absence of conditional heteroscedasticity could not be rejected for the relationship between active cultural participation and health and well-being measures, i.e., having controlled for the set of covariates mentioned in the previous section, respondents were identical. There are several reasons why this condition is demanding. First, the problem of correlation of error terms arose naturally, as a consequence of the same individuals being observed more than once, i.e., in successive waves of panel data. Second, there was a potential omitted variables problem, as other covariates might have influenced measures of health. Third, the problem of reverse causality arose not only as health status offered greater advantage to those participating more frequently, but also because those who enjoyed good health were more likely to participate more frequently. Thus, cultural attendance has an endogenous nature with respect to health. Fourth, unobservable individual effects – such as genetic and non-genetic factors – would be correlated with both health status and cultural attendance. Finally, error would be inherent in the measurement of health – as it depended solely on self-report.

In order to address these issues, first, the Breusch-Pagan test [22] was conducted to verify whether the null hypothesis for lack of conditional heteroscedasticity could be rejected. Then, in the next step the Durbin-Wu-Hausman test [23] was conducted to establish whether a more efficient random effects estimator could be used or the problem of omitted variables required the use of a fixed effects estimator. Having confirmed that a random effects model was likely to produce biased estimates with respect to both considered outcomes, the panel data regression with fixed effects estimator [24] was applied. To circumvent the endogeneity problem, instrumental variables (IV) [25] were used. The instrumental variables chosen are presented below.

Analysis was computed using Stata 14, applying the standard *logit*, *regress* and *xtreg* commands. The *xtivreg2* command [26], which implements IV and generalised method of moments (GMM) estimation for fixed-effects and first-differences panel data models with potential endogenous regressors were also used for panel data analysis.

## Results

The analysis of association between cultural attendance and health, modelled through the simple OLS regression, confirmed a positive relationship with SRH, but not with PHQ-15 (Table 1, columns “pooled OLS estimator”). However, as mentioned previously, standard OLS estimates can yield unbiased results only when the hypothesis of lack of conditional heteroscedasticity is not rejected for the relationship between cultural participation and a health measure, i.e., having controlled for the set of covariates mentioned in section 2, respondents were identical. The Breusch-Pagan test confirmed that in all models analysed, the pooled OLS estimator produced inferior results with respect to the random effects estimator (χ^2^_SRH_ = 10930.4, *p*-value_SRH_ < 0.001; *χ*^2^_*PHQ − 15*_ = 11945.1, *p*-value_PHQ-15_ < 0.001) implying that random effects at individual level were present. The results of Durbin-Wu-Hausman test also showed that a random effects model was likely to yield biased estimates and thus, should be rejected (*χ*^2^_*SRH*_ = 145.8, *p*-value_SRH_ < 0.001; *χ*^*2*^_*PHQ − 15*_ = 240.1 p-value_PHQ-15_ < 0.001). Consequently, with respect to both outcomes considered, the fixed effects estimator was used to estimate coefficients for the panel regression models.

**Table 1:** Estimates from regression of culture on health

	SRH			PHQ-15		
Independent variable	pooled OLS estimator	fixed-effects model	fixed-effects model with IV	pooled OLS estimator	fixed-effects model	fixed-effects model with IV
Constant	3.915*** (0.154)	---	---	2.588** (0.427)	---	---
Cultural attendance	0.045** (0.006)	0.022*** (0.006)	0.020 (0.106)	0.001 (0.020)	−0.013 (0.019)	−0.056 (0.353)
*Control variables*						
Gender (ref. female)	−0.158*** (0.012)	---	---	1.193*** (0.036)	---	---
Age	−0.022*** (0.000)	−0.014*** (0.002)	−0.014*** (0.002)	0.088*** (0.001)	−0.024*** (0.008)	−0.024** (0.008)
Education	0.022*** (0.000)	0.005 (0.005)	0.004 (0.005)	−0.046* (0.010)	0.027 (0.018)	0.028 (0.018)
Unemployed (ref. others)	0.002 (0.012)	−0.022 (0.027)	−0.022 (0.027)	−0.193* (0.046)	−0.190** (0.084)	−0.193* (0.087)
ln (income)	0.202** (0.021)	0.036** (0.016)	0.037* (0.017)	−0.474** (0.071)	0.001 (0.059)	0.004 (0.064)
*Instruments*	No	No	Yes	No	No	Yes
Observations	51,962	51,962	51,962	50,661	50,661	50,661

*** *p* < 0.001; ** *p* < 0.01; * *p* < 0.05; robust and clustered standard errors of estimates in parentheses; *IV* instrumental variables

The results of the fixed effects model (Table 1, column SRH, fixed effects model) indicated that there was positive association between cultural participation and self-reported health. Each additional attendance at the cinema, theatre or concert coincided with a 0.022 point higher rate of self-reported health on a six point scale. This relation, despite being very weak, however proved highly significant, owing to the very large sample size. A different picture emerged from analysis of the interrelation between cultural attendance and PHQ-15. In the fixed effect panel regression on the PHQ-15 scale, cultural attendance was non-significant. This implied that even an associational relationship between the two was unlikely.

These steps covered all issues, with the exception of reverse causality and endogeneity. To address this, instrumental variables were employed [25]. This, to the best of our knowledge, it was the first attempt to causally link attendance at cultural events and health using instrumental variables. Search for strong instruments was focussed on variables, highly correlated with cultural participation and exogenous with respect to health status. First, following the reasoning of d’Hombres et al. [27], that community level variables can be useful instruments, average attendance at cultural events in a given NUTS 2 region and the size of place of residence were chosen. It was assumed that participation by neighbouring households represented availability and trends in the local community and thus, directly influenced individual cultural behaviour. Additionally, the state of individual health was, logically, by no means an influence on regional attendance rate.

Second, book ownership was considered as an instrument for cultural participation. More culturally oriented people were considered likely to own more books. Also, influence of health status on possession of books seems highly unlikely, since individual libraries are built over long periods and should, therefore, be immune to direct health status effects. In order to further limit reverse causality, library size was dichotomized, separating individuals with large from those with small libraries, with ownership of up to 100 or over 100 books classified into different groups. Thus, any effect attributed to direct acquisition of books in response to changing health status was accounted for. Third, in order to account for the economic aspect of cultural engagement, affordability for attendance at cultural events was also considered, as it should, logically, strongly influence actual attendance. This should be unrelated to health status as this instrument detected non-attendance for reasons of financial pressure, as opposed to lack of interest in culture without the financial hardship.

Therefore, the cultural attendance variable in both panel regressions was initially instrumented by two dichotomous variables: number of books in a household (0 = no more than 100 books, 1 = more than 100 books) and financial hardship, on cinema, theatre, opera or other concert going in the last year (0 = no, 1 = yes) and one continuous variable, representing the average cultural participation in Polish NUTS 2 regions given the size of place of residence. Given the representative nature of Social Diagnosis data in NUTS 2 regions and inclusion of the size of place of residence in the sampling design, the averages represented reliable estimates for cultural attendance in the neighbourhood.

All three instruments were proved against classical over-identification using the Hansen J statistic (the counterpart of the Sargan [28] statistic for estimation with robust standard errors) in the regression with the PHQ-15 scale (χ^2^_PHQ − 15_ = 5.451, p-value_PHQ-15_ = 0.065). However, for SRH, exclusion of one instrument – the impact of financial hardship on cinema, theatre, concert going – was necessary in order to confirm sufficiently strong coherence for the set of instruments [29] yielding the Hansen J statistic *χ *^*2*^_*SRH*_ = 1.039 (*p*-value_SRH_ = 0.308). Additionally, in both fixed-effect panel regressions, the instruments passed the weak identification test (the Kleibergen-Paap Wald F statistic – counterpart to the Cragg-Donald Wald F statistic for estimation with robust standard errors – 32.31 and 29.98, for the SRH and PHQ-15 panel regressions, respectively). Following Stock and Yogo [30], for models with two and three instruments the 5 % critical values of the test for weak instruments were 19.93 and 22.30, allowing rejection of the hypothesis that bias from the instrumental variable estimator (relative to the OLS, in our case – fixed effect estimator) exceeded the desired 10 % for worst-case bias. The weak instrument hypothesis could, therefore, be rejected, implying that IVs used in the panel regressions achieved the required control for reverse causality and endogeneity problems. Estimates from the fixed effect panel regressions with instrumental variables are presented in Table 1, columns “Fixed-effect model with IV”.^2^

Fixed-effects panel regressions with IV for both SRH and PHQ-15 indicated that attendance at cultural events was not significant for improvement in either self-reported or physical health. With respect to PHQ-15, the significance of estimates was in line with both OLS and fixed-effects estimators. For SRH, the positive influence of cultural participation suggested by the OLS and fixed effects estimators, without account for endogeneity and reverse causality, was compromised. Therefore, results recommend rejection of both H1 and H2. They did not confirm that more frequent cultural event attendance had either positive causal influence on self-reported health (H1) or that any decrease in prevalence of the symptoms of ill health (reflecting physical health) was derived from cultural attendance (H2), in the Polish population.

## Discussion

Influence of cultural participation proved non-significant in the longitudinal study of Polish individuals for either self-reported health (SRH) or actual physical health, as reflected by the somatic symptom scale (PHQ-15). These results contrast to the positive association between cultural participation and SRH which is regularly reported [1, 3, 5, 31–33], but are substantiated by the adding of the longitudinal perspective and causal link. No evidence was found to suggest a positive impact of cultural participation on PHQ-15, which represented the physical health indicator. Contrary to Konlaan et al. [33] and Bygren et al. [7], who, using a Swedish cohort study found positive influences of leisure time activity on survival and attendance at cultural events on cancer mortality, neither correlation-based nor causally significant links between cultural attendance and physical health were found. It should be stated, however, that since a substantially different instrument for health assessment was used, this comparison should be treated with caution. Konlaan et al. [33] and Bygren et al. [7] assessed health through the lens of mortality rates, while our instrument reflected something closer to quality of life and psychological distress [13]. Nevertheless, the study reported here provides little evidence to support promotion of attendance at cultural events with the intention of improving population health in Polish population. Similar results were reported by Węziak-Białowolska [34] for Swiss population. However, these findings do not contest that participation in other types of more active, cultural and creative, arts-related activities may be beneficial to health.

Regardless of developments with the use of panel data and causal inference, this study is also threatened by other challenges. Firstly, as there is only one questionnaire item in the study related to cumulative cultural attendance, we were not able to disentangle passive from active involvement in their potential contributions to good health. This may bias our results as research shows that these two types of cultural activities may differ in their health association [35–37]. So, using a single indicator limits the reliability of our instrument and thus, results. We recognise that there is a disjuncture between our approach and the theoretically supported approach, however, this reflects the best approach given the data available. Second, there is long-standing debate on the understanding of perceived health measures, in particular, the self-rated health question [38–40] and its use in longitudinal studies [41]. Although this measure is believed to provide useful information on overall individual health status, concerns about its interpretation and self-health-assessment in general are florid. These may be highly dependent on the contextual framework, including the cultural and biographical background of an individual, but also rely on social characteristics such as social class or standard of living. This study attempted to control for them but as only the Polish population was under investigation, control for inter-cultural differences was beyond the scope.

The fact that our investigation was restricted to the Polish situation creates a third limitation. While nothing is established about any possible peculiarity of Polish culturally related health behaviour (since this topic has, so far, mainly remained un-investigated), the results do not permit extrapolation to other populations. The authors believe, however, that this study may guide health promotion policy with respect to cultural events.

## Conclusions

We showed the relation between attendance at cultural events and self-reported health could only be confirmed as associational. Therefore, this study provided little justification to encourage use of passive cultural participation as a measure of health promotion (improvement). Our study did not confirm any identifiable benefit to physical health from passive participation in culture. Future research should investigate the causative influence of active participation in creative activities on health outcomes as, in contrast to passive attendance, it may be influential.

## Abbreviations

GMM, generalized method of moments; IV, instrumental variables; OLS, ordinary least squares; PHQ-15, patient health questionnaires; SRH, self-reported health

## Appendix

**Table 2:** Patient Health Questionnaire (PHQ-15). In the past month: 0 - I did not suffer; 1- I suffered less than 15 days; 2 - I suffered at least for one half of the month

1. strong headaches	9. accelerated heartbeat (palpitation)
2. stomach pains or flatulence	10. shivers or convulsions
3. pain or tension in the neck or arm muscles	11. pressure on the bladder and more frequent urination
4. chest or heart pains	12. a feeling tiredness not associated with work
5. dry mouth or throat	13. constipation
6. attacks of excessive sweating	14. nosebleeds
7. shortness of breath	15. sudden changes of blood pressure
8. Pain in your arms, legs or joints (knees, hips, etc.)

**Table 3:** Estimates from first stage regression of control and instrumental variables on cultural attendance (estimation with attrition weights)

Independent variable	SRH	PHQ-15
Age	−0.004* (0.002)	−0.005** (0.002)
Education	0.004 (0.004)	0.003 (0.004)
Unemployed (ref. others)	−0.047 (0.024)	−0.057* (0.026)
ln (income)	0.063*** (0.015)	0.063*** (0.014)
average participation in culture events in a given NUTS 2 region	0.860*** (0.107)	0.860*** (0.107)
financial hardship applies to cinema, theatre and concert going (ref. no)	---	−0.056*** (0.012)
number of books in a household (ref. no more than 100 books)	0.043* (0.043)	0.040* (0.019)
Observations	51,962	50,661

*** *p* < 0.001; ** *p* < 0.01; * *p* < 0.05; robust standard errors of estimates in parentheses; *IV* instrumental variables

**Table 4:** Comparison of model estimates – with and without attrition weights and multiple imputations – Self-rate health (SRH)

	pooled OLS estimator			fixed-effects model			fixed-effects model with IV		
Independent variable	No weighs, no imputations	Attrition weighs, no imputations	No weighs, multiple imputations (10)	No weighs, no imputations	Attrition weighs, no imputations	No weighs, multiple imputations (10)	No weighs, no imputations	Attrition weighs, no imputations	No weighs, multiple imputations (10)
Constant	3.910*** (0.154)	**3.915*** (0.154)**	3.879*** (0.081)	4.336*** (0.128)	**---**	4.100*** (0.005)	---	**---**	---
Cultural participation	0.045** (0.006)	**0.045** (0.006)**	0.045* (0.005)	0.022*** (0.006)	**0.022*** (0.006)**	0.036*** (0.005)	0.019 (0.107)	**0.020 (0.106)**	---
*Control variables*									
Gender (ref. female)	−0.157*** (0.011)	**−0.158*** (0.012)**	−0.112** (0.010)	---	**---**	---	---	**---**	---
Age	−0.029*** (0.000)	**−0.022*** (0.000)**	−0.029*** (0.001)	−0.014*** (0.002)	**−0.014*** (0.002)**	−0.016*** (0.002)	−0.014*** (0.002)	**−0.014*** (0.002)**	---
Education	0.022*** (0.000)	**0.022*** (0.000)**	0.016* (0.001)	0.005 (0.004)	**0.005 (0.005)**	0.005* (0.002)	0.004 (0.005)	**0.004 (0.005)**	---
Unemployed (ref. others)	0.002 (0.011)	**0.002 (0.012)**	0.039 (0.016)	−0.021 (0.025)	**−0.022 (0.027)**	−0.001 (0.022)	−0.022 (0.027)	**−0.022 (0.027)**	---
ln (income)	0.202** (0.021)	**0.202** (0.021)**	0.202** (0.010)	0.036* (0.015)	**0.036** (0.016)**	0.096*** (0.011)	0.037* (0.017)	**0.037* (0.017)**	---
*Instruments*	No	**No**	No	No	**No**	No	Yes	**Yes**	---
Observations	51,962	**51,962**	144,199	51,962	**51,962**	144,199	51,962	**51,962**	---

*** *p* < 0.001; ** *p* < 0.01; * *p* < 0.05; robust and clustered standard errors of estimates in parentheses; *IV* instrumental variables; models presented in the paper are emboldened

To facilitate comparisons we repeated the estimates from Table 1 (in bold)

**Table 5:** Comparison of model estimates - with and without attrition weights and multiple imputations - GHQ-15

	pooled OLS estimator			fixed-effects model			fixed-effects model with IV		
Independent variable	No weighs, no imputations	Attrition weighs, no imputations	No weighs, multiple imputations (10)	No weighs, no imputations	Attrition weighs, no imputations	No weighs, multiple imputations (10)	No weighs, no imputations	Attrition weighs, no imputations	No weighs, multiple imputations (10)
Constant	2.605** (0.430)	**2.588** (0.427)**	3.648* (0.328)	5.686*** (0.464)	**---**	5.564*** (0.347)	---	**---**	---
Cultural participation	0.001 (0.020)	**0.001 (0.020)**	0.009 (0.012)	−0.013 (0.023)	**−0.013 (0.019)**	−0.001 (0.019)	−0.054 (0.609)	**−0.056 (0.353)**	---
*Control variables*									
Gender (ref. female)	1.119*** (0.037)	**1.193*** (0.036)**	0.830** (0.056)	---	**---**	---	---	**---**	---
Age	0.088*** (0.001)	**0.088*** (0.001)**	0.085*** (0.003)	−0.024** (0.008)	**−0.024*** (0.008)**	0.014 (0.008)	−0.024** (0.008)	**−0.024** (0.008)**	---
Education	−0.046* (0.010)	**−0.046* (0.010)**	−0.052* (0.008)	0.028 (0.016)	**0.027 (0.018)**	−0.023 (0.012)	0.028 (0.018)	**0.028 (0.018)**	---
Unemployed (ref. others)	−0.195* (0.046)	**−0.193* (0.046)**	−0.294 (0.065)	−0.191* (0.090)	**−0.190** (0.084)**	−0.226* (0.095)	−0.193* (0.087)	**−0.193* (0.087)**	---
ln (income)	−0.477** (0.072)	**−0.474** (0.071)**	−0.501* (0.042)	−0.002 (0.055)	**0.001 (0.059)**	−0.230*** (0.044)	0.005 (0.063)	**0.004 (0.064)**	---
*Instruments*	No	**No**	No	No	**No**	No	Yes	**Yes**	---
Observations	50,661	**50,661**	144,481	50,661	**50,661**	144,481	50,661	**50,661**	---

*** *p* < 0.001; ** *p* < 0.01; * *p* < 0.05; robust and clustered standard errors of estimates in parentheses; *IV* instrumental variables; models presented in the paper are emboldened

To facilitate comparisons we repeated the estimates from Table 1 (in bold)

## Footnotes

1 The NUTS classification (Nomenclature of territorial units for statistics) is a hierarchical system for division of the economic territory of the EU to collect, develop and harmonise the European regional statistics. Several layers are distinguishable. NUTS 0 correspond to countries, NUTS 1 are major socio-economic regions, NUTS 2 are basic regions of regional policy. NUTS 3 are small regions for specific diagnosis.

2 The estimates from the first stage regression are presented in the appendix.
